# Bis[[aqua­(1*H*-imidazo[4,5-*f*][1,10]phenanthroline-κ^2^ *N* ^6^,*N* ^7^)cadmium]bis­(μ-pyridine-2,3-dicarboxyl­ato)-κ^3^ *N*,*O* ^2^:*O* ^3^;κ^3^ *O* ^3^:*N*,*O* ^2^]

**DOI:** 10.1107/S160053681200579X

**Published:** 2012-02-17

**Authors:** Li-Juan Chen, Ming-Xing Yang, Hua Huang, Xiao-Hua Chen, Shen Lin

**Affiliations:** a College of Chemistry and Materials Science, Fujian Normal University, Fuzhou, Fujian 350007, People’s Republic of China

## Abstract

In the title compound, [Cd_2_(C_7_H_3_NO_4_)_2_(C_13_H_8_N_4_)_2_(H_2_O)_2_], the Cd^II^ ion is six-coordinated by two N atoms from a 1*H*-imidazo[4,5-*f*][1,10]phenanthroline (IP) ligand, one N atom and one O atom from a pyridine-2,3-dicarboxyl­ate (pdc) ligand, one O atom from another pdc ligand and one water mol­ecule in a distorted octa­hedral geometry. Two Cd^II^ ions are bridged by a pair of pdc ligands, forming a centrosymmetric dinuclear structure. Neighboring dinuclear units are linked by the coordinated water mol­ecules through O—H⋯N and O—H⋯O hydrogen bonds, forming a layer parallel to (011). The layers are further linked into a three-dimensional network through N—H⋯O hydrogen bonds. π–π inter­actions between the IP ligands further stabilize the supra­molecular structure [centroid–centroid distances = 3.579 (3), 3.686 (3), 3.710 (3), 3.766 (3) and 3.841 (3) Å].

## Related literature

For general background to metal–organic coordination polymers, see: Wang, Chen, Gao *et al.* (2010 ▶); Wang *et al.* (2011 ▶). For related structures, see: Liu *et al.* (2009 ▶, 2011 ▶); Wang, Chen, Wang *et al.* (2010 ▶).
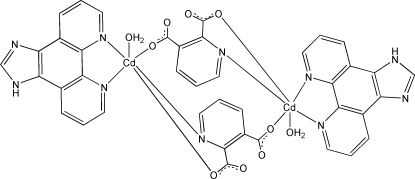


## Experimental

### 

#### Crystal data

[Cd_2_(C_7_H_3_NO_4_)_2_(C_13_H_8_N_4_)_2_(H_2_O)_2_]
*M* *_r_* = 1031.51
Triclinic, 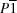

*a* = 7.474 (4) Å
*b* = 10.214 (5) Å
*c* = 12.641 (7) Å
α = 80.333 (6)°
β = 72.974 (5)°
γ = 80.170 (5)°
*V* = 902.0 (8) Å^3^
*Z* = 1
Mo *K*α radiation
μ = 1.26 mm^−1^
*T* = 293 K
0.20 × 0.20 × 0.15 mm

#### Data collection

Rigaku Mercury CCD diffractometer
Absorption correction: multi-scan (*CrystalClear*; Rigaku, 2002 ▶) *T*_min_ = 0.710, *T*_max_ = 1.000
5733 measured reflections
3028 independent reflections
2814 reflections with *I* > 2σ(*I*)
*R*_int_ = 0.022

#### Refinement

*R*[*F* ^2^ > 2σ(*F* ^2^)] = 0.032
*wR*(*F* ^2^) = 0.084
*S* = 1.06
3028 reflections
280 parameters
H-atom parameters constrained
Δρ_max_ = 0.51 e Å^−3^
Δρ_min_ = −0.47 e Å^−3^

### 

Data collection: *CrystalClear* (Rigaku, 2002 ▶); cell refinement: *CrystalClear*; data reduction: *CrystalClear*; program(s) used to solve structure: *SHELXS97* (Sheldrick, 2008 ▶); program(s) used to refine structure: *SHELXL97* (Sheldrick, 2008 ▶); molecular graphics: *DIAMOND* (Brandenburg, 1999 ▶); software used to prepare material for publication: *SHELXL97*.

## Supplementary Material

Crystal structure: contains datablock(s) I, global. DOI: 10.1107/S160053681200579X/hy2513sup1.cif

Structure factors: contains datablock(s) I. DOI: 10.1107/S160053681200579X/hy2513Isup2.hkl

Additional supplementary materials:  crystallographic information; 3D view; checkCIF report

## Figures and Tables

**Table 1:** Hydrogen-bond geometry (Å, °)

*D*—H⋯*A*	*D*—H	H⋯*A*	*D*⋯*A*	*D*—H⋯*A*
N3—H3*A*⋯O4^i^	0.86	1.86	2.691 (5)	164
O5—H5*B*⋯O2^ii^	0.84	1.80	2.633 (5)	174
O5—H5*C*⋯N4^iii^	0.84	1.99	2.799 (5)	161

Symmetry codes: (i) 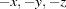; (ii) 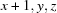; (iii) 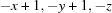.

**Fig. 1.**
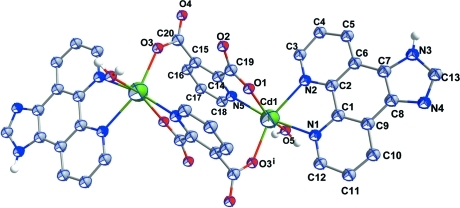
Molecular structure of the title complex, showing 50% probability displacement ellipsoids. H atoms bonded to C atoms are omitted. [Symmetry code: (i) -x, -y, 1-z.]

**Fig. 2.**
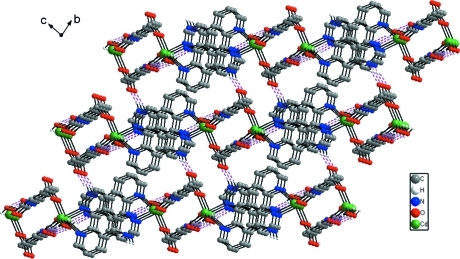
The three-dimensional structure formed through hydrogen bonds and π–π stacking interactions.

## supplementary crystallographic information

### Comment

Metal organic frameworks (MOFs) have received much attention due to their intriguing structural diversity and tremendous potential applications in catalysis, optics, electrics, magnetism, and so on (Wang, Chen, Gao *et al.*, 2010; Wang *et al.*, 2011). When used to build supramolecular architectures, 1*H*-imidazol(4,5-f)(1,10-phenanthroline) (IP) may have profound influence on the final structrues of MOFs, owing to its excellent coordinating ability, large conjugated system and the presence of the N—H group (Liu *et al.*, 2009, 2011; Wang, Chen, Wang *et al.*, 2010). The title compound was prepared based on IP and pyridine-2,3-dicarboxylic acid (H_2_pdc) ligands.

The asymmetric unit of the title compound consists of one half of the dimeric complex, which lies about an inversion center. The Cd^II^ atom is six-coordinated by two N atoms from an IP ligand, one N atom and one O atom from a pdc ligand, one O atom from another pdc ligand and one O atom from an aqua group (Fig. 1). The two Cd^II^ atoms are bridged by a pair of pdc ligands, forming a centrosymmetric dinuclear structure.

It is noteworthy that various hydrogen bonds are observed in the title compound. (*a*) O—H···N and O—H···O hydrogen bonds involve the coordinated water molecule O5, the imidazole N4 and carboxylate O2 atoms (Table 1). These two hydrogen bonds connect neighboring dinuclear units, forming a layer. (*b*) An N—H···O hydrogen bond between the imine group N3—H3A and the carboxylate O4 atom (Table 1) extends the layers into a three-dimensional network (Fig. 2). The offset π–π interactions between the IP ligands, with centroid–centroid distances ranging from 3.579 (3) to 3.841 (3) Å, further stabilize the supramolecular structure.

### Experimental

A mixture of CdCl_2_.2.5H_2_O (0.3 mmol), H_2_pdc (0.3 mmol) and IP(0.3 mmol) in 8 ml distilled water was placed in a 18 ml Teflon-lined Parr acid digestion bomb and heated for 3 d at 433 K under autogenous pressure. Slow cooling of the reaction mixture to room temperature gave colorless prism crystals (yield: 30% based on Cd).

### Refinement

The water H atoms were located in a difference Fourier map and fixed in refinement with O—H = 0.84 Å and *U*_iso_(H) = 1.2*U*_eq_(O). Other H atoms were placed geometrically and refined as riding, with C—H = 0.93 and N—H = 0.86 Å and with *U*_iso_(H) = 1.2*U*_eq_(C, N).

### Crystal data

**Table:**

[Cd_2_(C_7_H_3_NO_4_)_2_(C_13_H_8_N_4_)_2_(H_2_O)_2_]	*Z* = 1
*M**_r_* = 1031.51	*F*(000) = 512
Triclinic, *P*1	*D*_x_ = 1.899 Mg m^−^^3^
Hall symbol: -P 1	Mo *K*α radiation, λ = 0.71073 Å
*a* = 7.474 (4) Å	Cell parameters from 2480 reflections
*b* = 10.214 (5) Å	θ = 3.2–27.5°
*c* = 12.641 (7) Å	µ = 1.26 mm^−^^1^
α = 80.333 (6)°	*T* = 293 K
β = 72.974 (5)°	Prism, colorless
γ = 80.170 (5)°	0.20 × 0.20 × 0.15 mm
*V* = 902.0 (8) Å^3^	

### Data collection

**Table:**

Rigaku Mercury CCD diffractometer	3028 independent reflections
Radiation source: fine-focus sealed tube	2814 reflections with *I* > 2σ(*I*)
Graphite monochromator	*R*_int_ = 0.022
Detector resolution: 14.6306 pixels mm^-1^	θ_max_ = 25.0°, θ_min_ = 2.0°
ω scans	*h* = −8→8
Absorption correction: multi-scan (*CrystalClear*; Rigaku, 2002)	*k* = −12→12
*T*_min_ = 0.710, *T*_max_ = 1.000	*l* = −15→13
5733 measured reflections	

### Refinement

**Table:**

Refinement on *F*^2^	Primary atom site location: structure-invariant direct methods
Least-squares matrix: full	Secondary atom site location: difference Fourier map
*R*[*F*^2^ > 2σ(*F*^2^)] = 0.032	Hydrogen site location: inferred from neighbouring sites
*wR*(*F*^2^) = 0.084	H-atom parameters constrained
*S* = 1.06	*w* = 1/[σ^2^(*F*_o_^2^) + (0.0501*P*)^2^ + 0.6574*P*] where *P* = (*F*_o_^2^ + 2*F*_c_^2^)/3
3028 reflections	(Δ/σ)_max_ < 0.001
280 parameters	Δρ_max_ = 0.51 e Å^−^^3^
0 restraints	Δρ_min_ = −0.47 e Å^−^^3^

### Special details

**Table:**

Geometry. All e.s.d.'s (except the e.s.d. in the dihedral angle between two l.s. planes) are estimated using the full covariance matrix. The cell e.s.d.'s are taken into account individually in the estimation of e.s.d.'s in distances, angles and torsion angles; correlations between e.s.d.'s in cell parameters are only used when they are defined by crystal symmetry. An approximate (isotropic) treatment of cell e.s.d.'s is used for estimating e.s.d.'s involving l.s. planes.
Refinement. Refinement of *F*^2^ against ALL reflections. The weighted *R*-factor *wR* and goodness of fit *S* are based on *F*^2^, conventional *R*-factors *R* are based on *F*, with *F* set to zero for negative *F*^2^. The threshold expression of *F*^2^ > σ(*F*^2^) is used only for calculating *R*-factors(gt) *etc*. and is not relevant to the choice of reflections for refinement. *R*-factors based on *F*^2^ are statistically about twice as large as those based on *F*, and *R*- factors based on ALL data will be even larger.

### Fractional atomic coordinates and isotropic or equivalent isotropic displacement parameters (Å^2^)

**Table:**

	*x*	*y*	*z*	*U*_iso_*/*U*_eq_	
Cd1	0.26858 (4)	0.16248 (2)	0.30239 (2)	0.02641 (12)	
N1	0.2719 (4)	0.3800 (3)	0.2016 (2)	0.0242 (6)	
N2	0.2600 (4)	0.1574 (3)	0.1127 (3)	0.0294 (7)	
N3	0.2066 (5)	0.4622 (3)	−0.2285 (3)	0.0344 (8)	
H3A	0.1959	0.4151	−0.2761	0.041*	
N4	0.2306 (5)	0.6433 (3)	−0.1566 (3)	0.0322 (7)	
N5	0.2571 (4)	−0.0638 (3)	0.3757 (3)	0.0268 (7)	
O1	−0.0331 (3)	0.1178 (2)	0.3352 (2)	0.0305 (6)	
O2	−0.1954 (4)	−0.0475 (3)	0.3424 (3)	0.0467 (8)	
O3	−0.2560 (4)	−0.2618 (3)	0.5495 (2)	0.0339 (6)	
O4	−0.1475 (4)	−0.3605 (3)	0.3967 (2)	0.0416 (7)	
O5	0.5869 (4)	0.1286 (3)	0.2421 (2)	0.0368 (7)	
H5B	0.6524	0.0746	0.2781	0.044*	
H5C	0.6230	0.2047	0.2285	0.044*	
C1	0.2593 (5)	0.3962 (3)	0.0950 (3)	0.0213 (7)	
C2	0.2494 (5)	0.2759 (4)	0.0488 (3)	0.0246 (8)	
C3	0.2495 (7)	0.0489 (4)	0.0717 (4)	0.0420 (10)	
H3B	0.2560	−0.0331	0.1162	0.050*	
C4	0.2294 (7)	0.0509 (5)	−0.0348 (4)	0.0483 (12)	
H4A	0.2227	−0.0281	−0.0600	0.058*	
C5	0.2197 (6)	0.1693 (4)	−0.1011 (3)	0.0379 (10)	
H5A	0.2065	0.1721	−0.1723	0.045*	
C6	0.2298 (5)	0.2876 (4)	−0.0618 (3)	0.0255 (8)	
C7	0.2241 (5)	0.4174 (4)	−0.1214 (3)	0.0266 (8)	
C8	0.2397 (5)	0.5300 (4)	−0.0796 (3)	0.0251 (8)	
C9	0.2572 (5)	0.5218 (4)	0.0316 (3)	0.0241 (8)	
C10	0.2717 (5)	0.6327 (4)	0.0795 (3)	0.0291 (8)	
H10A	0.2711	0.7176	0.0389	0.035*	
C11	0.2865 (6)	0.6147 (4)	0.1857 (3)	0.0338 (9)	
H11A	0.2974	0.6867	0.2186	0.041*	
C12	0.2849 (5)	0.4865 (4)	0.2443 (3)	0.0304 (8)	
H12A	0.2935	0.4752	0.3173	0.036*	
C13	0.2101 (6)	0.5969 (4)	−0.2415 (4)	0.0382 (10)	
H13A	0.1987	0.6517	−0.3064	0.046*	
C14	0.0898 (5)	−0.1061 (3)	0.3911 (3)	0.0223 (7)	
C15	0.0544 (5)	−0.2352 (3)	0.4378 (3)	0.0231 (7)	
C16	0.2012 (5)	−0.3232 (4)	0.4680 (3)	0.0270 (8)	
H16A	0.1830	−0.4108	0.4990	0.032*	
C17	0.3719 (5)	−0.2801 (4)	0.4519 (3)	0.0346 (9)	
H17A	0.4706	−0.3379	0.4717	0.042*	
C18	0.3952 (5)	−0.1499 (4)	0.4060 (3)	0.0342 (9)	
H18A	0.5111	−0.1207	0.3957	0.041*	
C19	−0.0594 (5)	−0.0030 (4)	0.3535 (3)	0.0248 (8)	
C20	−0.1331 (5)	−0.2867 (3)	0.4612 (3)	0.0251 (8)	

### Atomic displacement parameters (Å^2^)

**Table:**

	*U*^11^	*U*^22^	*U*^33^	*U*^12^	*U*^13^	*U*^23^
Cd1	0.03043 (17)	0.02316 (17)	0.02730 (19)	−0.00802 (11)	−0.01124 (12)	0.00299 (12)
N1	0.0313 (16)	0.0207 (15)	0.0204 (16)	−0.0054 (13)	−0.0088 (13)	0.0032 (13)
N2	0.0404 (18)	0.0206 (15)	0.0279 (18)	−0.0083 (14)	−0.0117 (14)	0.0034 (14)
N3	0.0405 (19)	0.041 (2)	0.0244 (17)	−0.0069 (15)	−0.0158 (15)	0.0023 (15)
N4	0.0382 (18)	0.0333 (18)	0.0243 (18)	−0.0107 (15)	−0.0082 (14)	0.0041 (15)
N5	0.0254 (15)	0.0240 (16)	0.0310 (18)	−0.0077 (13)	−0.0098 (13)	0.0053 (14)
O1	0.0305 (14)	0.0234 (14)	0.0375 (16)	−0.0064 (11)	−0.0130 (12)	0.0063 (12)
O2	0.0381 (16)	0.0333 (16)	0.076 (2)	−0.0140 (13)	−0.0342 (16)	0.0161 (16)
O3	0.0323 (14)	0.0474 (17)	0.0247 (15)	−0.0164 (12)	−0.0064 (12)	−0.0027 (13)
O4	0.0490 (17)	0.0441 (17)	0.0419 (18)	−0.0124 (14)	−0.0175 (14)	−0.0179 (15)
O5	0.0304 (14)	0.0301 (14)	0.0506 (18)	−0.0043 (12)	−0.0175 (13)	0.0048 (13)
C1	0.0225 (17)	0.0208 (17)	0.0184 (18)	−0.0057 (14)	−0.0036 (13)	0.0032 (14)
C2	0.0218 (17)	0.0294 (19)	0.0225 (19)	−0.0063 (15)	−0.0048 (14)	−0.0023 (16)
C3	0.066 (3)	0.023 (2)	0.039 (3)	−0.010 (2)	−0.015 (2)	−0.0001 (19)
C4	0.078 (3)	0.035 (2)	0.039 (3)	−0.015 (2)	−0.018 (2)	−0.012 (2)
C5	0.048 (2)	0.042 (2)	0.028 (2)	−0.011 (2)	−0.0103 (18)	−0.0083 (19)
C6	0.0297 (19)	0.031 (2)	0.0188 (19)	−0.0092 (16)	−0.0079 (15)	−0.0032 (16)
C7	0.0263 (18)	0.037 (2)	0.0177 (19)	−0.0069 (16)	−0.0080 (15)	−0.0006 (17)
C8	0.0274 (18)	0.0268 (19)	0.0191 (19)	−0.0081 (15)	−0.0055 (14)	0.0061 (16)
C9	0.0230 (17)	0.0259 (18)	0.024 (2)	−0.0058 (14)	−0.0084 (15)	0.0010 (16)
C10	0.037 (2)	0.0213 (18)	0.028 (2)	−0.0056 (16)	−0.0095 (17)	0.0016 (16)
C11	0.048 (2)	0.027 (2)	0.028 (2)	−0.0107 (18)	−0.0090 (18)	−0.0086 (18)
C12	0.043 (2)	0.0273 (19)	0.024 (2)	−0.0056 (17)	−0.0148 (17)	−0.0013 (16)
C13	0.042 (2)	0.041 (2)	0.030 (2)	−0.0084 (19)	−0.0175 (19)	0.0169 (19)
C14	0.0243 (17)	0.0233 (17)	0.0188 (18)	−0.0049 (14)	−0.0054 (14)	0.0001 (15)
C15	0.0287 (18)	0.0220 (17)	0.0210 (19)	−0.0059 (14)	−0.0090 (15)	−0.0028 (15)
C16	0.034 (2)	0.0204 (18)	0.027 (2)	−0.0039 (15)	−0.0114 (16)	0.0024 (16)
C17	0.028 (2)	0.032 (2)	0.042 (2)	0.0004 (17)	−0.0141 (17)	0.0034 (19)
C18	0.0234 (19)	0.037 (2)	0.042 (2)	−0.0077 (16)	−0.0137 (17)	0.0089 (19)
C19	0.0256 (18)	0.0250 (19)	0.0243 (19)	−0.0073 (15)	−0.0087 (15)	0.0029 (16)
C20	0.0299 (19)	0.0200 (17)	0.028 (2)	−0.0065 (15)	−0.0141 (16)	0.0035 (16)

### Geometric parameters (Å, º)

**Table:**

Cd1—O3^i^	2.246 (3)	C3—C4	1.394 (6)
Cd1—O5	2.260 (3)	C3—H3B	0.9300
Cd1—O1	2.281 (3)	C4—C5	1.355 (6)
Cd1—N5	2.347 (3)	C4—H4A	0.9300
Cd1—N1	2.369 (3)	C5—C6	1.402 (5)
Cd1—N2	2.427 (3)	C5—H5A	0.9300
N1—C12	1.322 (5)	C6—C7	1.411 (5)
N1—C1	1.359 (4)	C7—C8	1.378 (5)
N2—C3	1.323 (5)	C8—C9	1.437 (5)
N2—C2	1.342 (5)	C9—C10	1.404 (5)
N3—C13	1.361 (5)	C10—C11	1.358 (5)
N3—C7	1.391 (5)	C10—H10A	0.9300
N3—H3A	0.8600	C11—C12	1.392 (6)
N4—C13	1.300 (5)	C11—H11A	0.9300
N4—C8	1.387 (5)	C12—H12A	0.9300
N5—C18	1.340 (5)	C13—H13A	0.9300
N5—C14	1.342 (4)	C14—C15	1.387 (5)
O1—C19	1.255 (4)	C14—C19	1.523 (5)
O2—C19	1.234 (4)	C15—C16	1.397 (5)
O3—C20	1.253 (5)	C15—C20	1.512 (5)
O4—C20	1.237 (4)	C16—C17	1.368 (5)
O5—H5B	0.8400	C16—H16A	0.9300
O5—H5C	0.8400	C17—C18	1.377 (6)
C1—C9	1.394 (5)	C17—H17A	0.9300
C1—C2	1.466 (5)	C18—H18A	0.9300
C2—C6	1.432 (5)		
			
O3^i^—Cd1—O5	95.63 (10)	C6—C5—H5A	120.1
O3^i^—Cd1—O1	103.29 (9)	C5—C6—C7	126.2 (3)
O5—Cd1—O1	157.08 (10)	C5—C6—C2	117.1 (4)
O3^i^—Cd1—N5	103.40 (11)	C7—C6—C2	116.7 (3)
O5—Cd1—N5	91.49 (10)	C8—C7—N3	105.3 (3)
O1—Cd1—N5	71.77 (9)	C8—C7—C6	123.7 (3)
O3^i^—Cd1—N1	86.07 (11)	N3—C7—C6	130.9 (3)
O5—Cd1—N1	89.37 (10)	C7—C8—N4	111.1 (3)
O1—Cd1—N1	104.56 (10)	C7—C8—C9	120.9 (3)
N5—Cd1—N1	170.36 (10)	N4—C8—C9	127.9 (3)
O3^i^—Cd1—N2	154.99 (11)	C1—C9—C10	118.6 (3)
O5—Cd1—N2	88.28 (11)	C1—C9—C8	117.7 (3)
O1—Cd1—N2	80.06 (10)	C10—C9—C8	123.7 (3)
N5—Cd1—N2	101.17 (10)	C11—C10—C9	119.3 (4)
N1—Cd1—N2	69.25 (10)	C11—C10—H10A	120.4
C12—N1—C1	118.5 (3)	C9—C10—H10A	120.4
C12—N1—Cd1	123.0 (2)	C10—C11—C12	118.9 (3)
C1—N1—Cd1	118.4 (2)	C10—C11—H11A	120.6
C3—N2—C2	118.3 (3)	C12—C11—H11A	120.6
C3—N2—Cd1	124.9 (3)	N1—C12—C11	123.3 (3)
C2—N2—Cd1	116.6 (2)	N1—C12—H12A	118.3
C13—N3—C7	105.0 (3)	C11—C12—H12A	118.3
C13—N3—H3A	127.5	N4—C13—N3	115.2 (4)
C7—N3—H3A	127.5	N4—C13—H13A	122.4
C13—N4—C8	103.3 (3)	N3—C13—H13A	122.4
C18—N5—C14	118.7 (3)	N5—C14—C15	122.5 (3)
C18—N5—Cd1	126.9 (2)	N5—C14—C19	115.6 (3)
C14—N5—Cd1	114.3 (2)	C15—C14—C19	121.9 (3)
C19—O1—Cd1	117.3 (2)	C14—C15—C16	117.7 (3)
C20—O3—Cd1^i^	135.5 (2)	C14—C15—C20	124.8 (3)
Cd1—O5—H5B	122.1	C16—C15—C20	117.5 (3)
Cd1—O5—H5C	106.4	C17—C16—C15	119.8 (3)
H5B—O5—H5C	110.6	C17—C16—H16A	120.1
N1—C1—C9	121.4 (3)	C15—C16—H16A	120.1
N1—C1—C2	117.3 (3)	C16—C17—C18	119.0 (4)
C9—C1—C2	121.2 (3)	C16—C17—H17A	120.5
N2—C2—C6	122.1 (3)	C18—C17—H17A	120.5
N2—C2—C1	118.2 (3)	N5—C18—C17	122.4 (3)
C6—C2—C1	119.7 (3)	N5—C18—H18A	118.8
N2—C3—C4	123.5 (4)	C17—C18—H18A	118.8
N2—C3—H3B	118.2	O2—C19—O1	125.9 (3)
C4—C3—H3B	118.2	O2—C19—C14	116.0 (3)
C5—C4—C3	119.2 (4)	O1—C19—C14	118.0 (3)
C5—C4—H4A	120.4	O4—C20—O3	124.7 (3)
C3—C4—H4A	120.4	O4—C20—C15	117.2 (3)
C4—C5—C6	119.8 (4)	O3—C20—C15	117.7 (3)
C4—C5—H5A	120.1		

Symmetry code: (i) −*x*, −*y*, −*z*+1.

### Hydrogen-bond geometry (Å, º)

**Table:**

*D*—H···*A*	*D*—H	H···*A*	*D*···*A*	*D*—H···*A*
N3—H3*A*···O4^ii^	0.86	1.86	2.691 (5)	164
O5—H5*B*···O2^iii^	0.84	1.80	2.633 (5)	174
O5—H5*C*···N4^iv^	0.84	1.99	2.799 (5)	161

Symmetry codes: (ii) −*x*, −*y*, −*z*; (iii) *x*+1, *y*, *z*; (iv) −*x*+1, −*y*+1, −*z*.
